# The Pharmacodynamics of Antipsychotic Drugs in Women and Men

**DOI:** 10.3389/fpsyt.2021.650904

**Published:** 2021-04-09

**Authors:** Mary V. Seeman

**Affiliations:** Department of Psychiatry, University of Toronto, Toronto, ON, Canada

**Keywords:** sex/gender, antipsychotics, pharmacodynamics, pharmacokinetics, response, side effects

## Abstract

**Background:** Animal and human experiments have confirmed sex differences in the expression of hepatic enzymes that metabolize antipsychotic drugs and that may, in this way, be partly responsible for the clinical sex/gender differences observed in the efficacy and tolerability of antipsychotic treatment.

**Aim:** The aim of this mini review is to synthesize the literature on the pharmacodynamics of male/female differential response to antipsychotic drugs.

**Method:** Relevant search terms were used to search for pre-clinical and human trials and analysis of antipsychotic differential drug response and occurrence/severity of adverse effects in women and men.

**Results:** The search found that sex influences drug response *via* the amount of a given drug that enters the brain and the number of neurotransmitter receptors to which it can bind. Consequently, sex partly determines the efficacy of a specific drug and its liability to induce unwanted effects. There are other factors that can overshadow or enhance the dimorphic effect of sex, for instance, the host's age, hormonal status, diet and life style as well as the molecular structure of the drug and its dose, and the method of its administration. Most of all, the host's individual genetics affects each step of a drug's pharmacodynamics.

**Conclusion:** On average, women's psychotic symptoms respond to antipsychotic drugs at doses lower than men's. This means that many women may be *de facto* overdosed and, thus, experience unnecessary adverse effects. That being said, factors such as genetics and age probably determine drug response and tolerability to a greater degree than do biological sex or gender social roles.

## Introduction

The term, pharmacodynamics, refers to how a drug works in the human body to produce its effects, both the intended effects for which a drug is prescribed and the unwanted effects that often occur. A drug's mechanism of action depends on the nature of the molecule itself, on its daily dose, and on how much of it reaches the sites in the body where it can exert its effect. It also depends on the particulars of the host—age, sex, frailty, and genetic factors. This minireview addresses the pharmacodynamics of antipsychotic medications (AP) as it is affected by the sex of the host. AP are drugs used to treat psychotic conditions; there are many in common usage, with more being continuously developed in the hope of improving efficacy and reducing the toll of adverse effects. As will be explained, the pharmacodynamics of each of these many drugs is not identically affected by sex, though the mechanism by which these drugs reduce psychotic symptoms is similar. They all block brain neurotransmitters at their receptor sites on neuronal membranes.

Beaulieu et al. stated in 2015 [(1), p. 14], “no clinically approved antipsychotic drugs exist to date that are not D2 receptor blockers.” As a result, this review will focus discussion of the effect of sex on metabolism and response to antipsychotics targeting the D2 receptor.

### Neurotransmitter Receptors and Sex

In all species, dopamine (DA) receptors play a critical role in arousal, sensory processing, and motivation, as well as in motor behavior (2). These receptors are transmembrane proteins that transmit signals by binding to DA molecules released into the synaptic cleft by nearby DA-releasing cells. Once the DA attaches to the receptor, it enables the postsynaptic cell to start the process of sending out an array of signals to neighboring cells in the brain. Such signals ultimately influence sensation, motivation, cognition, and motor activity as well as affecting the secretion of hormones. There are four main pathways in the brain that transmit DA signals: the nigrostriatal pathway involved in motor control, the mesolimbic involved in incentive and motivation, the mesocortical, which influences learning and memory, and the tuberoinfundibular, which controls prolactin production.

Animal experiments show that dopamine signaling, which includes the synthesis of dopamine, its release and reuptake, and subsequent postsynaptic responses, are all sex-dependent effects, some of which (but not all) are influenced by gonadal hormone levels in the plasma (3).

In order to reverse psychotic symptoms in AP-responsive patients, it has been calculated that ~60% of striatal D2 receptors on postsynaptic cells need to be occupied by the antidopaminergic ligand (4–6). Approximately 80% occupation of these receptors, however, results in unacceptable extrapyramidal side effects (5), and this threshold may be modulated by sex. For instance, Okita et al. (7) have shown that smoking cigarettes affects the D2 receptor to different degrees in men and women. Animal experiments have demonstrated sex effects on receptor uptake of radioactive ligands (8). One pre-clinical study has shown sex effects on the upregulation of D2 receptors after treatment with haloperidol or clozapine (9). In terms of effects in humans, Kaasinen et al. (10) found that women had higher D2-like receptor binding potentials than men, especially in the left and right anterior cingulate cortex.

Receptor occupancy is determined by PET imaging of a radioactive antidopaminergic ligand. When this was modeled in 70 patients using olanzapine as the ligand, Eugene and Masiak (11) found that women needed a smaller oral dose than men to achieve the ideal occupancy. The average daily dose of olanzapine required by women was estimated to be 10 mg compared to 20 mg for men. Estimated required doses of all antipsychotics can be potentially determined using this method (12).

The amount of DA transporter, which is the protein that transports the dopamine in the synaptic cleft back into the presynaptic cell and, thus, regulates synaptic DA availability, is also higher in women compared to men (13), which could, at least theoretically, influence the amount of dopamine transmitted from neuron to neuron.

Importantly, D2 receptor availability has been reported to vary with fluctuations in the level of plasma sex steroid hormones, across the menstrual cycle for example (14). This suggests that it also varies over the course of pregnancy, and is affected by the advent of labor and delivery and the menopause. It may also be impacted by contraceptives, hormone replacement therapy, IVF treatment, or other forms of hormonal intervention.

The determination of receptor occupancy can be used not only to determine drug dosages required for AP efficacy but also to estimate the dose likely to induce adverse effects, such as hyperprolactinemia (15) and total side effect burden (16).

Many antipsychotics bind to more than one neurotransmitter receptor. Clozapine, for instance, blocks adrenergic α1 and α_2_ receptors, the histamine H_1_ receptor, and most muscarinic and glutaminergic receptors (17). Though DA receptors seem to be the most important with respect to drug efficacy against delusions and hallucinations, there are other contenders for that role. Women have been reported to have higher 5-HT1A receptor numbers than men in certain brain regions (18) and also to have more cortical muscarinic receptors (19). Clozapine may be exceptionally effective because of its influence on a large number of brain receptors. The downside, however, is that each receptor blockade can induce its own adverse effects.

Several radioactive ligands can be used in imaging studies to visualize and quantify receptor occupancy at receptor sites. In the clinic, therapeutic drug monitoring or measuring the plasma levels of drugs, can be used as a proxy for neurotransmitter occupancy (20). But the accuracy of this method will depend on cerebral flow rate [higher in females than males (21)] and the pharmacokinetics of the drug, as discussed in the next section.

### Pharmacokinetics and Sex

The sexes differ with respect to absorption, distribution, metabolism, and elimination of most drugs (22, 23). Men and women, on average, differ in levels of gastric acidity, degree of intestinal motility, body weight and distribution of adipose tissue, blood volume, liver enzymes (mainly the cytochrome P450 series), and renal excretion rates, each of these factors affecting plasma drug levels in separate ways (Table 1).

**Table 1:** Sex/gender and antipsychotic effects.

**Phenomenon**	**Higher in women**	**Higher in men**
Gastric absorption	X	
Lipid storage	X	
Amount entering brain	X	
Number of concomitant drugs	X	
Amount of P-glycoprotein	X	
Kidney excretion rate		X
Psychiatric side effects		X
Physical side effects	X	

### Absorption

The absorption of a drug depends to some degree on the characteristics of the drug but also, if the drug is taken by mouth, on characteristics of the gastrointestinal environment. Gastric acidity is lower in women than in men and gastric emptying time is slower. Intestinal transit time is also slower. The solubility of drugs can be affected by the composition of bile acid which also differs between men and women. When these effects are taken together, women tend to absorb drugs more thoroughly than men (22). Other things being equal, this means that the onset of action of an orally-administered drug will be quicker in women than in men.

### Distribution

A drug's volume of distribution is determined by the host's body weight, percentage of adipose tissue (for lipophilic drugs), blood volume (for water soluble drugs), the degree of local blood flow to relevant body sites, and the extent of the drug's binding to blood proteins. Women are characterized by lower body weight, but drug dosing in adults is not currently adjusted for body weight. AP drugs accumulate in adipose tissue. Since women's bodies are composed of proportionally more adipose tissue than the bodies of men, the half-life of these drugs and the risk of high drug concentrations should weight be suddenly lost is significantly increased. Clinically, it also means that stopping a lipophilic drug such as an AP will lead to a more rapid relapse of symptoms in men (whose store of adipose tissue is smaller) than in women. For those on depot injections, it means that intervals between injections can safely, on average, be longer in women than in men (24).

Drugs bind to plasma proteins in the blood stream and only the quantity that is unbound can pass through the blood–brain barrier and reach the brain. In general, the binding capacity of plasma proteins seems to be lower in women than in men, leading to more free drug and, therefore, more availability at relevant neuroreceptor sites. The administration of two drugs at the same time, both with high protein binding properties, causes one of them to be displaced, thus increasing its free fraction and central nervous system activity. This is important because, on average, women with schizophrenia, are prescribed more concomitant medications (antidepressants, mood stabilizers, pain killers, sedatives, and contraceptives or hormone replacements) than men are (24).

### Elimination

Drugs are eliminated from the blood stream *via* hepatic, renal, or pulmonary routes and, to a minor degree, through sweat, tears, and breast milk. The kidney, *via* glomerular filtration, is the major organ of excretion and this rate is faster, on average, in men than in women (22).

Hepatic clearance is a function of liver blood flow and hepatic enzyme activity. Hepatic blood flow is lower in women than in men. More importantly, there are hormone-dependent sex differences in the amounts of available metabolizing enzymes (although this mainly depends on the host's individual genetics). The amount of P-glycoprotein, which regulates the biliary excretion of some drugs, is 2-fold lower in women than in men (22).

### Metabolism

The metabolism of drugs is accomplished, in part, by cytochrome P450 (CYP) enzymes, some of which, due to hormonal influences, show differential activity in men and women. The isozymes CYP2D6, CYP3A4, CYP1A1/2, and CYP2C19 are responsible for the metabolism of most AP drugs. No sex differences have been described in the base activity of CYPD6, but pregnancy has been reported to induce this enzyme (25), as can the use of contraceptives and hormone replacement therapy (26). It must be remembered, however, that the gene for CYP2D6 has over 100 allele variants, so that different individuals are able to express marked differences in its enzymatic activity (27). Nevertheless, it means that the dose of AP mainly metabolized by CYP2D6 (risperidone, thioridazine, perphenazine, fluphenazine, zuclopenthixol, haloperidol, and chlorpromazine) may need to be increased during pregnancy.

Women show less CYP2C19 enzymatic activity than men. A region on the promoter region of the CYP2C19 is down-regulated by estrogen derivatives, which also means that the enzyme is inhibited by oral contraceptives (28).

Reports of male/female divergence with respect to CYP1A2 activity are not consistent, probably because of a 200-fold interindividual difference, but the bulk of the evidence leans toward less activity in women than in men. The activity of this enzyme is known to vary with menstrual cycle and pregnancy status (29). CYP1A2, the main metabolizing enzyme for olanzapine and clozapine, shows relatively reduced activity in women so that the average doses of these two drugs need to be reduced in women to avoid adverse effects. CYP1A2 activity is increased in smokers and is inhibited by some antidepressants (especially fluvoxamine), some first generation AP, and also valproic acid (30–33).

The strongest gender difference among the CYP isozymes involves CYP3A4, which is hyperactive in women of reproductive age. This means potentially lower concentrations of haloperidol, perphenazine, aripiprazole, ziprasidone, quetiapine, and risperidone in women than in men on the same dose. CYP3A4 activity is higher in Caucasians than in African Americans, and in Caucasian women than in Asian women (34).

Pharmacokinetics is also affected by phase 2 glucuronidation and UDP glucuronosyl transferases (UGTs), which are activated by estrogen (29). The influence of sex hormones is, therefore, potentially substantive for the pharmacokinetics of AP drugs as a whole, the net effect being that the amount of free drug that enters the brain is higher in women than in men (35). On the other hand, the genetics of the host may overshadow sex differences.

### Side Effects Resulting From the Pharmacodynamics of Antipsychotics

Antipsychotics interact with many transmitters, with target receptors not only in the brain but throughout the body, creating the many known adverse effects that interfere with the effectiveness of schizophrenia treatment. Because of differences in pharmacokinetics and pharmacodynamics, clinically relevant adverse drug reactions differ between men and women (36). Changing levels of sex steroids throughout life, especially at critical times such as puberty, pregnancy, menopause and aging, affect not only drug absorption, distribution, metabolism, and elimination but also the binding to neurotransmitter receptors. There are also important effects of exogenous hormones—hormonal contraceptives, hormone replacement at menopause, transgender therapy, and performance enhancing steroids. Interactions with other drugs taken at the same time play a role, as do modes of drug delivery (oral, injection, depot).

The foregoing determines not only drug efficacy, but also side effects. In a cross-sectional study, Iversen et al. (37) found that over 75% of individuals taking AP report adverse effects—extrapyramidal effects, sexual symptoms, sedation, and weight gain. Twice as many women as men described these effects as being severe.

An interesting method that can contrast side effects in men and women is text mining electronic patient records. Using this method, Sørup et al. (38) found 55 potential adverse effects with significantly different frequencies in men and women. Twenty were more frequent in men and 35 more frequent in women. Psychiatric side effects were noted more in male records whereas physical effects were seen more often in female records.

### Tardive Dyskinesia

Tardive dyskinesia (TD) continues to be an adverse effect affecting considerable numbers of individuals on AP, up to 20% of those on 2nd generation drugs (39), which are known to induce relatively few extrapyramidal symptoms.

Older age is the major risk factor here. While the risk of TD is reportedly higher in women, this may largely be due to larger numbers of aging women in the populations studied. The other associated risks for TD are the presence of a mood disorder, a history of marked extrapyramidal symptoms and use of anticholinergic drugs. If it is true that AP doses prescribed for women are higher than they need to be, thus leading to extra dopamine receptors being occupied (40), then a female susceptibility to TD may be more apparent than real.

### Agranulocytosis

Like TD, agranulocytosis has been considered to be more prevalent in women than in men but a recent meta-analysis of 36 studies of clozapine-treated patients (0.4% had developed agranulocytosis) found no significant difference between the sexes. There is a higher prevalence in older patients. This and the relatively small number of women in most study samples may explain why women have been considered to be at greater risk for agranulocytosis (41).

### Cardiac Arrythmia

Several AP drugs cause prolongation of the QT interval (beginning of the Q wave till the end of the T wave on the electrocardiogram); some APs do this more than others (42).

QT prolongation can sometimes lead to the potentially fatal arrhythmia called torsades de pointes (TdP). Of the newer AP, ziprasidone confers the highest risk while aripiprazole appears to be safest. Risk goes up proportionately to dose although as many as 75% of TdP cases occur at therapeutic doses. Age over 65 is a risk factor, as is polypharmacy with other QT-prolonging drugs. There is also a genetic susceptibility (43).

The incidence of drug-induced TdP is higher in women than in men, probably because adult women, in general, show a longer QT interval than adult men, an effect of female hormones that has been first observed after the onset of puberty. The effect is heightened during pregnancy (44, 45).

### Metabolic Effects

The adverse metabolic risks (obesity, type 2 diabetes, dyslipidemia, hypertension) associated with second generation antipsychotics are well-known, and contribute to the high rate of cardiovascular morbidity and pre-mature death in the schizophrenia population. Female schizophrenia patients are diagnosed with metabolic diseases at higher rates than males, which may be attributable to the average woman being *de facto* overdosed. This is most likely to happen with olanzapine and clozapine because of the pharmacokinetics noted earlier (46, 47).

In a recent review, Castellani et al. (48) found that the majority of clinical studies indicated that women on AP gained more weight than men, and were more at risk for metabolic syndrome. The pathway to metabolic side-effects induced especially by second generation AP remains incompletely understood. It is known that all AP increase peripheral catecholamines in a dose dependent fashion, but with variation among the different AP. The quantity of peripheral epinephrine, norepinephrine, and dopamine in peripheral tissues is increased by different AP in a manner that is consistent with their metabolic liability (49).

To summarize what is known about metabolic dimorphism, gender effects are somewhat inconsistent. This is probably because polymorphisms in genes coding for drug-metabolizing enzymes, transporters, and receptors lead to a great variety of individual reactions, irrespective of sex.

### Hyperprolactinemia

According to their effect on prolactin, antipsychotics can be classified into two groups: relatively prolactin-sparing and prolactin-raising. The difference depends on how strongly the drugs bind, and for how long, to dopamine receptors. In the text mining study referred to earlier, the results showed that multiple hyperprolactinemia-related adverse effects (sexual difficulties, acne, hirsutism, galactorrhea, gynecomastia, menorrhagia, infertility) tend to occur together in women (38). Whether breast cancer can be included as a potential adverse effect of AP-induced hyperprolactinemia in women is an important issue that continues to remain controversial (50).

The incidence of breast cancer is higher in women with schizophrenia than in other women but this is probably due to confounding by indication (other aspects of schizophrenia and its sequelae being responsible for breast cancer, not high prolactin levels) (51). These other aspects are low parity, obesity, extensive alcohol use and smoking. The high mortality rate of breast cancer in women with schizophrenia can probably also be attributed to factors associated with illness (low rate of cancer screening, cognitive and motivational factors that interfere with diagnosis and treatment) (52, 53). Osteoporosis, more prevalent in women than in men, may also result from hyperprolactinemia, but its elevated prevalence in schizophrenia may again be mainly attributable other factors, such as to low rates of screening and failure to receive or adhere to treatment (54, 55).

The more minor side effects of AP-induced hyperprolactinemia (acne, hirsutism, infertility, menstrual, and sexual difficulties) while, to clinicians, may appear insignificant when compared to the symptoms of psychosis, can, nevertheless, exert major influence on the quality of life in patients with schizophrenia (56).

In general, judging from hospital statistics, the adverse and sometimes toxic effects of AP are more prevalent in women than in men (40). AP are not alone in this respect. Women experience all adverse drug reactions nearly twice as often as men. Of 86 drugs evaluated, adverse reactions for most were more severe in women than in men. This has been attributed to the differences in pharmacokinetics, which results, sometimes, in the overdosing of women (57, 58). Women suffer more allergic reactions to drugs than men do (59, 60), and they more readily report their side effects (57). From the first year of treatment with AP, women lead men in reporting unwanted effects (61).

## Conclusion

This review of the pharmacodynamics of antipsychotics in men and women suggests that the biology of sex and the impact of gender-associated social roles (adherence to prescriptions, readiness to report adverse effects, life style) both affect antipsychotic drug efficacy and tolerability. On average, compared to men, women's psychotic symptoms respond to many of these drugs at lower doses. This means that, in many cases, standard doses are too high for women and cause unnecessary adverse effects. Other factors, however, such as individual genetics and chronological age, play an even greater role than sex in determining dose efficacy and extent of adverse events.

## Author Contributions

The author confirms being the sole contributor of this work and has approved it for publication.

## Conflict of Interest

The author declares that the research was conducted in the absence of any commercial or financial relationships that could be construed as a potential conflict of interest.
